# Comparable clinical outcomes and redislocation rates after isolated medial patellofemoral ligament reconstruction in patients with and without borderline anatomical risk factors for patellar instability

**DOI:** 10.1002/jeo2.70928

**Published:** 2026-09-29

**Authors:** Alessio Maione, Simone Romano, Pierrenzo Pozzi, Matteo D. Parmigiani, Alessandra Menon, Paolo Ferrua, Filippo Calanna, Riccardo Compagnoni, Eva Usellini, Andrea Mambretti, Massimo Berruto, Pietro S. Randelli

**Affiliations:** ^1^ ASST Centro Specialistico Ortopedico Traumatologico Gaetano Pini‐CTO, 1° Clinica Ortopedica Milan Italy; ^2^ Residency Program in Orthopedics and Traumatology University of Milan Milan Italy; ^3^ ASST Centro Specialistico Ortopedico Traumatologico Gaetano Pini‐CTO, Traumatologia Sportiva Milan Italy; ^4^ ASST Centro Specialistico Ortopedico Traumatologico Gaetano Pini‐CTO, Ortopedia Traumatologia d'Urgenza Milan Italy; ^5^ Department of Clinical and Community Sciences, Graduate School of Health Statistics and Biometrics University of Milan Milan Italy; ^6^ Department of Biomedical Sciences for Health, Laboratory of Applied Biomechanics University of Milan Milan Italy; ^7^ ASST Centro Specialistico Ortopedico Traumatologico Gaetano Pini‐CTO, Week‐Surgery Milan Italy; ^8^ Department of Biomedical, Surgical and Dental Sciences University of Milan Milan Italy; ^9^ Department of Biomedical Sciences for Health, Research Center for Adult and Pediatric Rheumatic Diseases (RECAP‐RD) University of Milan Milan Italy

**Keywords:** knee surgery, MPFL reconstruction, patella alta, patellar instability, trochlear dysplasia

## Abstract

**Purpose:**

The purpose of this study was to compare mid‐ to long‐term clinical outcomes and redislocation rates after isolated medial patellofemoral ligament (MPFL) reconstruction in patients with and without borderline anatomical risk factors. It was hypothesized that isolated MPFL reconstruction would provide comparable outcomes regardless of borderline anatomical abnormalities.

**Methods:**

A retrospective comparative study was performed on 51 patients (55 knees) treated with isolated MPFL reconstruction for recurrent patellar instability between 2013 and 2023. Patients were divided into a standard group (Caton–Deschamps Index [CDI] < 1.2, tibial tubercle–trochlear groove [TT–TG] < 20 mm, absent or Type A trochlear dysplasia) and a borderline group (CDI 1.2–1.4, TT–TG 20–24 mm and/or Dejour Type B–D trochlear dysplasia without explosive J‐sign or large trochlear spur). Clinical outcomes were assessed using Knee injury and Osteoarthritis Outcome Score (KOOS), Kujala, Tegner and Visual Analogue Scale (VAS) scores at a mean follow‐up of 7.9 ± 3.9 years. Redislocations and complications were recorded.

**Results:**

Twenty‐five knees were included in the standard group and 30 in the borderline group. Both groups showed significant improvement in all clinical scores from baseline to final follow‐up (*p* < 0.001 for all). No significant differences were observed between groups at final follow‐up in KOOS (88.9 ± 10.3 vs. 86.1 ± 9.5; *p* = 0.159), Kujala (85.3 ± 18.8 vs. 86.9 ± 10.3; *p* = 0.713), Tegner (4.0 ± 1.1 vs. 3.9 ± 1.3; *p* = 0.765) or VAS scores (1.6 ± 1.1 vs. 2.1 ± 1.8; *p* = 0.260). Redislocation rates were comparable between groups (4.0% vs. 6.7%; *p* = 1.0). Within the borderline group, the cumulative burden of anatomical risk factors did not negatively affect outcomes.

**Conclusions:**

Isolated MPFL reconstruction provides reliable and durable mid‐ to long‐term outcomes in carefully selected patients with borderline anatomical risk factors, with clinical results and redislocation rates comparable to those observed in standard patients. These findings support a patient‐specific surgical approach, suggesting that additional bony procedures should be reserved for clearly pathological anatomical abnormalities rather than routinely performed in borderline cases.

**Level of Evidence:**

Level III.

## Graphical abstract

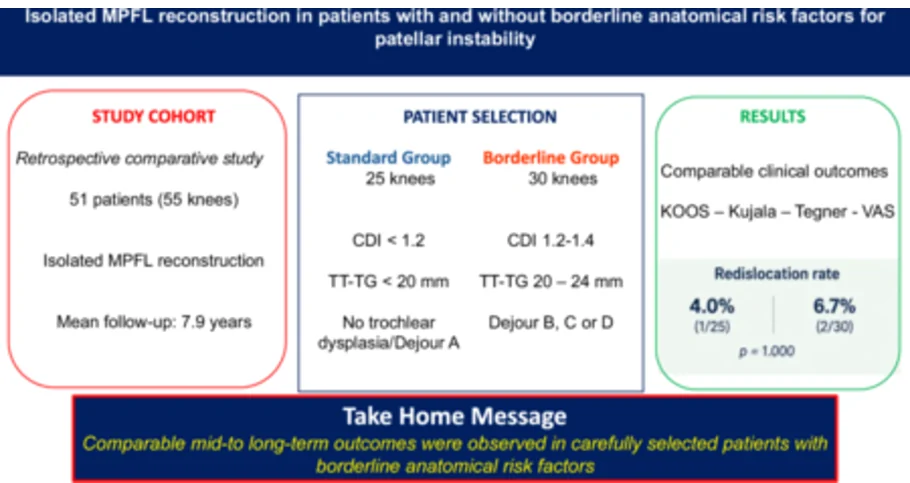

**Abbreviations**
CDI: Caton–Deschamps Index
CPM: continuous passive motion
CT: computed tomography
FU: follow‐up
Gr: gracilis
KOOS: Knee injury and Osteoarthritis Outcome Score
LARS: Ligament Advanced Reinforcement System
MCID: minimal clinically important difference
MPFL: medial patellofemoral ligament
MRI: magnetic resonance imaging
No: number
ROM: range of motion
SD: standard deviation
SPE: sagittal patellar engagement
St: semitendinosus
TT–TG: tibial tubercle–trochlear groove
VAS: Visual Analogue Scale

## INTRODUCTION

Patellofemoral instability is a common and clinically relevant condition, particularly affecting paediatric and adolescent populations [9, 19, 28, 42], with a higher incidence among physically active individuals and athletes. It encompasses a wide spectrum of clinical presentations, ranging from episodic subluxation to recurrent lateral patellar dislocation, often associated with pain, functional limitation and progressive cartilage damage [19]. Epidemiological studies report an overall incidence of approximately 23 per 100,000 individuals [15].

According to the 2024 European Society of Sports Traumatology, Knee Surgery and Arthroscopy (ESSKA) formal consensus [4], nonoperative treatment remains the standard of care after a first‐time patellar dislocation in the absence of osteochondral injury. However, recurrence rates may exceed 30% [37], particularly in younger patients and in those presenting with predisposing anatomical risk factors.

Patellofemoral instability is widely recognized as a multifactorial condition resulting from the interaction between soft tissue restraints, dynamic muscular control and bony anatomy. Trochlear dysplasia, patella alta and increased tibial tubercle–trochlear groove (TT–TG) distance represent the main anatomical risk factors [1], although other factors, such as coronal malalignment, may also contribute to patellofemoral instability [2, 7, 10, 34, 40]. In this context, the medial patellofemoral ligament (MPFL) plays a central role as the primary passive restraint to lateral patellar translation, contributing approximately 50%–60% of the restraining force in early knee flexion [16, 20]. Consequently, MPFL reconstruction has become the cornerstone of surgical treatment for recurrent patellar instability [8, 11, 22].

While surgical indications are relatively well established in the presence of severe anatomical abnormalities, the management of patients with borderline anatomical risk factors remains controversial. These patients present with values at or slightly above commonly accepted thresholds, making surgical decision‐making challenging. In particular, it remains unclear whether isolated MPFL reconstruction is sufficient or whether additional bony procedures should be systematically considered [5].

Current literature provides heterogeneous evidence. Several studies and systematic reviews [15, 17, 18, 30] have reported satisfactory outcomes following isolated MPFL reconstruction in the presence of mild to moderate anatomical abnormalities, whereas others have identified specific factors—such as patella alta or high‐grade trochlear dysplasia—as potential predictors of failure, especially when associated with additional clinical signs such as a high‐grade J‐sign [9, 37]. Unlike previous reports, the present study specifically focuses on carefully selected patients with borderline anatomical risk factors, reflecting a real‐world decision‐making algorithm aimed at avoiding unnecessary bony procedures. Patients were directly compared using uniform surgical indications and a mid‐ to long‐term follow‐up. These findings therefore provide additional evidence supporting a less aggressive surgical strategy.

Despite the growing body of literature, long‐term comparative data directly evaluating outcomes after isolated MPFL reconstruction in patients with and without borderline anatomical risk factors remain limited.

The purpose of this study was to evaluate whether patients with recurrent patellofemoral instability and borderline anatomical risk factors (P) treated with isolated MPFL reconstruction (I) achieve clinical outcomes and redislocation rates comparable to those of patients without borderline anatomical risk factors undergoing the same procedure (C) at mid‐ to long‐term follow‐up (T), with clinical scores and redislocation rate as the primary outcomes (O).

It was hypothesized that isolated MPFL reconstruction would provide comparable clinical outcomes and redislocation rates at mid‐ to long‐term follow‐up regardless of the presence of borderline anatomical risk factors.

## MATERIAL AND METHODS

### Study design and patient selection

A retrospective, monocentric comparative study was conducted in accordance with the Declaration of Helsinki and approved by the Regional Ethics Committee (Milano Area 2—Fondazione IRCCS Ca' Granda Ospedale Maggiore Policlinico, Milan, Italy; approval ID: 870_2021). Written informed consent was obtained from all participants.

Between January 2013 and December 2023, 111 patients (118 knees) underwent surgical treatment for recurrent lateral patellar dislocation at the study institution. Patients treated with isolated MPFL reconstruction were retrospectively identified. Fifty‐one patients (55 knees) met the inclusion criteria and were included in the final analysis.

Inclusion criteria were (1) recurrent patellar instability, defined as at least two documented lateral patellar dislocations or persistent instability after a first dislocation; (2) treatment with isolated MPFL reconstruction and (3) minimum follow‐up of 24 months.

Exclusion criteria included previous knee surgery, combined surgical procedures, concomitant ligament injuries, permanent patellar dislocation or fixed subluxation, explosive J‐sign (defined as lateral patellar translation greater than two quadrants in full extension during dynamic assessment) and the presence of a large trochlear spur, defined as a prominence >5 mm on magnetic resonance imaging (MRI). Patients with clinically significant coronal malalignment requiring corrective osteotomy were not considered candidates for isolated MPFL reconstruction and were therefore not included in the present study.

Patients were divided into two groups based on the presence of anatomical risk factors associated with patellofemoral instability:

- The standard group comprised patients with no major anatomical risk factors: Caton–Deschamps Index [6] (CDI) below 1.2, TT–TG distance below 20 mm and trochlear dysplasia absent or Dejour Type A only.
- The borderline group comprised patients presenting with at least one anatomical risk factor exceeding the standard range but considered suitable for isolated MPFL reconstruction according to the predefined decision‐making algorithm. Borderline parameters were defined as CDI 1.2–1.4, TT–TG distance 20–24 mm and/or Dejour Type B, C or D trochlear dysplasia. Isolated MPFL reconstruction was considered when these abnormalities were not associated with clinical and morphological features considered to warrant an additional bony procedure, particularly an explosive J‐sign, a large trochlear spur (>5 mm on MRI) or clinically significant coronal malalignment requiring corrective osteotomy.

Patients were classified as borderline if at least one of these criteria was present. The coexistence of multiple borderline parameters was not considered, per se, an indication for additional bony procedures, provided that none of the aforementioned exclusion criteria was present. One additional patient, whose static radiological parameters fell within the standard range, was allocated to the borderline group on the basis of a clinically relevant J‐sign that did not meet the explosive threshold (lateral patellar translation of two quadrants or less in full extension on dynamic assessment), reflecting a functional patellar maltracking not captured by static measurements.

The choice between hamstring tendon autograft and a synthetic graft (LARS) was based on patient‐specific characteristics and the availability and quality of the native hamstring tendons. The surgical technique, anatomical femoral and patellar fixation, and postoperative rehabilitation protocol remained unchanged regardless of the graft type.

When using the LARS graft, particular attention was paid to graft tensioning because of its greater stiffness and lower elasticity compared with autologous tendons. Excessive tensioning of a synthetic graft may overconstrain the patellofemoral joint, potentially reducing range of motion (ROM) and increasing postoperative anterior knee pain. Therefore, meticulous intraoperative tension adjustment was performed in all LARS cases to avoid overconstraint.

Demographic and radiological characteristics are summarized in Tables 1 and 2.

**Table 1:** Patient characteristics.^a^

	Overall	Standard group	Borderline group
No. of patients	51	22	29
No. of knees	55	25	30
Mean follow‐up, years	7.9 ± 3.9 [2.8–10.6]	7.8 ± 3.8 [3.1–9.7]	8.0 ± 4.1 [2.6–10.8]
Sex			
Male	16 (29.1)	8 (32.0)	8 (26.7)
Female	39 (70.9)	17 (68.0)	22 (73.3)
Graft			
St	39 (70.9)	20 (80.0)	19 (63.3)
Gr	1 (1.8)	0	1 (3.3)
LARS	15 (27.3)	5 (20.0)	10 (33.3)
Age at surgery, years	24.26 ± 10.20 [17.0–29.0]	24.52 ± 10.85 [17.0–29.0]	24.07 ± 9.86 [17.0–27.7]
Age at 1° dislocation, years	12.77 ± 2.83 [11.0–14.0]	13.57 ± 3.43 [11.0–14.0]	12.20 ± 2.20 [11.0–14.0]
No. of pre‐operative dislocations*	3.94 ± 1.98 [2.5–5.0]	3.14 ± 1.42 [2.0–5.0]	4.50 ± 2.15 [3.0–5.0]

Abbreviations: Gr, gracilis; LARS, Ligament Advanced Reinforcement System; No., number; Q1, first quartile; Q3, third quartile; SD, standard deviation; St, semitendinosus.

^a^ Data are expressed as mean ± SD, median [Q1–Q3] or number of patients (percentage).

* *p* value (standard vs. borderline): **0.013.**

**Table 2:** Risk factors overview.^a^

	Overall	Standard group	Borderline group	*p* value
CDI	1.09 ± 0.15 (range: 0.80–1.35)	1.03 ± 0.12 (range: 0.81–1.20)	1.15 ± 0.16 (range: 0.80–1.35)	0.005
SPE	44.21 ± 8.56 (range: 25–63)	46.70 ± 6.72 (range: 35–63)	42.13 ± 9.47 (range: 25–57)	0.077
TT–TG	17.1 ± 3.9 (range: 7–24)	14.9 ± 4.1 (range: 7–20)	19.0 ± 2.9 (range: 13–24)	<0.001
Trochlear dysplasia				<0.001*
Type A	18 (32.7)	16 (64.0)	2 (6.7)	
Type B	6 (10.9)	0	6 (20.0)	
Type C	12 (21.8)	0	12 (40.0)	
Type D	9 (16.4)	0	9 (30.0)	
No dysplasia	10 (18.2)	9 (36.0)	1 (3.3)	

Abbreviations: CDI, Caton–Deschamps Index; SD, standard deviation; SPE, sagittal patellar engagement; TT–TG, tibial tubercle–trochlear groove distance.

^a^ Data are expressed as mean ± SD, (range), except for trochlear dysplasia, which is expressed as *n* (%).

* *p* value refers to the distribution of trochlear dysplasia between groups. Statistically significant difference was set at *p *≤ 0.05.

### Radiological and clinical evaluation

Standardized radiographs were obtained preoperatively, postoperatively and at 12‐month follow‐up. Femoral tunnel positioning was assessed on true lateral postoperative radiographs using the grid method described by Servien et al., with tunnels located within the predefined anatomical region classified as anatomical and those outside this region as nonanatomical. In addition, the distance between the femoral tunnel and the ideal Schöttle point was measured in millimetres.

TT–TG distance was measured on computed tomography (CT) scans. Sagittal patellar engagement (SPE) was assessed on MRI and defined as the ratio between the length of patellar articular cartilage and the corresponding trochlear cartilage length measured at the level of the TG on sagittal imaging.

To determine intra‐ and interobserver reliability of radiographic assessments, the measurements were performed twice, at a 2‐month interval, by two orthopaedic surgeons (S.R. and P.P.) not involved in the surgical procedure. Intra‐ and interobserver reliability were evaluated using intraclass correlation coefficients (ICCs) with an absolute‐agreement, two‐way mixed‐effects model. According to the ICC values proposed by Koo and Li [23], the reliability was defined as poor (<0.5), moderate (0.5 ≤ ICC < 0.75), good (0.75 ≤ ICC < 0.90) or excellent (≥ 0.90). The ICC values demonstrated good to excellent reliability, with an intraobserver ICC of 0.92 and an interobserver ICC of 0.89, based on an absolute‐ agreement, two‐way mixed‐effects model.

Baseline was defined as the patient's clinical status immediately before surgery. Given the retrospective design of the study, baseline clinical data were retrieved retrospectively from the medical records and referred to the last preoperative clinical assessment.

Clinical outcomes were evaluated using the Knee injury and Osteoarthritis Outcome Score [35] (KOOS), Kujala score [24], Tegner Activity Scale and Visual Analogue Scale (VAS) for pain. These outcome measures were collected at baseline and at final follow‐up. Redislocation rates and complications were also recorded.

### Surgical technique

All procedures were performed by three senior knee surgeons using a standardized technique.

Before sterile draping, a careful dynamic clinical assessment was performed under anaesthesia to evaluate patellar tracking and to reassess the presence and severity of the J‐sign.

Graft selection was individualized based on patient characteristics, with autologous semitendinosus or gracilis tendons preferentially used in younger patients and a Ligament Advanced Reinforcement System (LARS) used in cases of higher mechanical demand or generalized ligamentous laxity. Fixation technique and hardware were identical across graft types.

A medial parapatellar approach was used to expose the superomedial patellar border. Two all‐suture anchors were placed, and the graft was secured at its midportion. The femoral insertion was identified under fluoroscopic guidance at the Schöttle point [38]. The graft was passed beneath the second layer and fixed in a femoral tunnel using a biocomposite interference screw at 60° of knee flexion [26] after confirming appropriate isometry and patellar tracking.

### Postoperative protocol

A knee brace was not routinely used postoperatively unless the patient was particularly apprehensive. Progressive weightbearing as tolerated was permitted starting on postoperative Day 1 with the assistance of crutches. Early restoration of knee motion was encouraged; a continuous passive motion (CPM) device (Kinetec; Kinetec S.A.S.) was used, beginning with an ROM of 0°–30° and daily increments of 5°, reaching 0°–90° by 2 weeks. From Weeks 2–4, full weight‐bearing and restoration of complete flexion were pursued. Quadriceps‐strengthening isometric exercises were initiated on postoperative Day 1; when quadriceps inhibition or difficulty with activation was present, neuromuscular electrical stimulation was employed. At 1 month postoperatively, provided no complications had occurred, stationary cycling and freestyle swimming were allowed. Running on level surfaces was permitted from 3 months, whereas return to pivoting sports was not recommended before 6 months.

### Statistical analysis

Statistical analysis was performed using R Statistical Software (Version 4.2.0; R Foundation for Statistical Computing). The Shapiro–Wilk normality test was used to evaluate the normal distribution of the sample. Continuous variables were expressed as the mean ± standard deviation (SD) or median and interquartile range [Q1–Q3], as appropriate. Categorical variables were expressed as numbers and frequencies, and between‐group differences were assessed using the *χ* ^2^ test or Fisher's exact test. The between‐group differences for continuous variables were evaluated with the unpaired Student's *t* test or Mann–Whitney *U* test, while the within‐group differences from baseline to final follow‐up for continuous variables were evaluated with the paired *t* test or Wilcoxon matched‐pairs signed‐rank test, according to the characteristics of the data distribution. Statistical significance was set at *p* < 0.05.

The comparison of the KOOS score between the standard and borderline groups was defined as the primary analysis; all other outcome comparisons, including secondary scores and subgroup analyses stratified by cumulative risk factor burden, were regarded as secondary and exploratory. Because the study was designed around a single primary endpoint, no formal adjustment for multiple comparisons was applied; the secondary and exploratory comparisons are reported descriptively and interpreted with the corresponding caution.

The potential influence of femoral tunnel positioning on clinical outcomes was assessed by comparing final KOOS, Kujala, Tegner and VAS scores between knees with anatomical and nonanatomical tunnel positioning using the Mann–Whitney *U* test. Spearman correlation analysis was additionally used to assess the relationship between distance from the ideal Schöttle point and clinical outcomes. Fisher's exact test was used for categorical comparisons.

Given the retrospective design, no a priori power calculation was performed. A post hoc analysis using the KOOS score as primary outcome (minimal clinically important difference [MCID] = 8 points, pooled SD = 9.9 points) demonstrated that the present sample (*n* = 25 and *n* = 30) achieved 83.4% power to detect a clinically meaningful between‐group difference (*α* = 0.05, two‐tailed), with a minimum detectable difference of 5.4 points. The observed between‐group difference at final follow‐up was 2.7 points, which was below both the predefined MCID and the calculated minimum detectable difference.

### Use of artificial intelligence

Generative artificial intelligence (ChatGPT; OpenAI) was used exclusively for English‐language editing, including correction of grammar and syntax, improvement of scientific wording, identification and removal of personal pronouns. All artificial intelligence (AI)‐assisted revisions were critically reviewed and approved by the authors.

## RESULTS

A total of 51 patients (55 knees) met the inclusion criteria and were included in the final analysis (Figure 1). The standard group comprised 25 knees, while the borderline group included 30 knees. The mean follow‐up was 7.9 ± 3.9 years (range: 2.0–12.7 years), with no significant difference between groups. Baseline demographic and clinical characteristics are summarized in Table 1.

**Figure 1:**
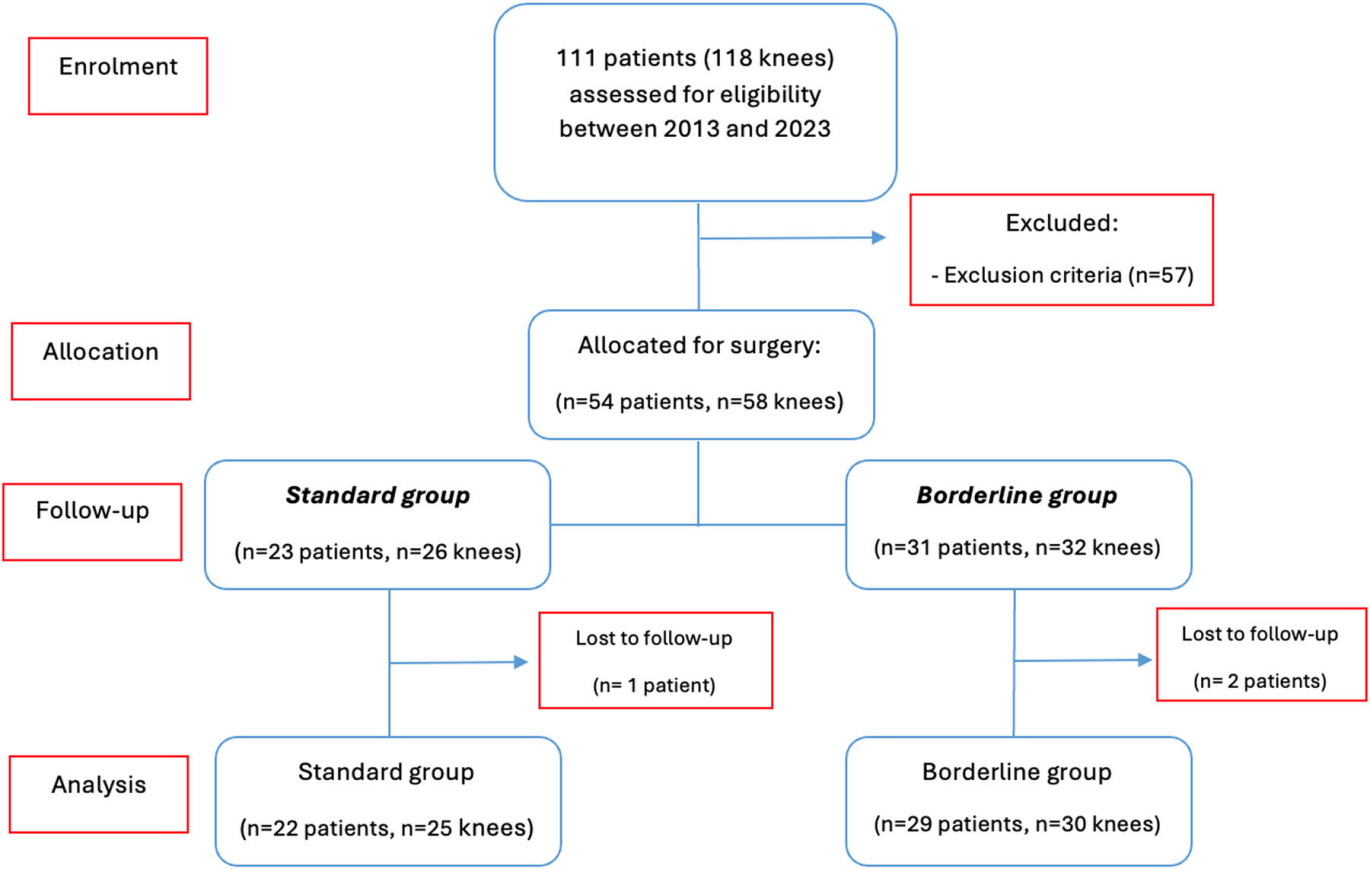
Flowchart of patient selection.

Both groups demonstrated significant improvement in all clinical outcome measures from baseline to final follow‐up (*p* < 0.001 for all), with no significant differences observed between the standard and borderline groups at final evaluation.

### Preoperative clinical and radiological characteristics

Baseline demographic characteristics were comparable between groups, including age at first dislocation and age at surgery.

The borderline group presented a significantly higher number of preoperative dislocation episodes compared with the standard group (4.5 ± 2.2 vs. 3.1 ± 1.4; *p *= 0.013).

SPE values were comparable between groups (standard group, 46.7 ± 6.7% vs. borderline group, 42.1 ± 9.5%; *p* = 0.077).

### Distribution of borderline risk factors and outcomes by cumulative risk factor burden

Within the borderline group, 9 knees (30.0%) had a CDI between 1.2–1.4 and 8 knees (26.7%) had a TT–TG distance between 20 and 24 mm. Regarding trochlear morphology, 15 knees (50.0%) presented Dejour Type B or D trochlear dysplasia, 12 (40%) Type C dysplasia, 2 (6.7%) Type A dysplasia and 1 knee (3.3%) had no trochlear dysplasia. Eleven knees (36.7%) fulfilled one criterion, 14 (46.7%) fulfilled two criteria and 4 (13.3%) fulfilled all three criteria. Notably, the four knees presenting both a CDI of 1.2–1.4 and a TT–TG distance of 20–24 mm also presented Dejour Type B or D trochlear dysplasia. One patient was included based on clinical assessment as described above. When stratified by the number of borderline criteria, clinical outcome scores at final follow‐up were similar across subgroups, with no trend toward poorer results as the number of criteria increased: mean KOOS was 84.2 ± 11.1, 87.8 ± 7.0 and 91.0 ± 5.0 in the one‐, two‐ and three‐criterion subgroups, respectively (*p* = 0.530); mean Kujala score was 87.0 ± 10.3, 87.9 ± 5.9 and 92.2 ± 4.2 (*p* = 0.477). Redislocations were infrequent in all subgroups (1 of 11, 1 of 14 and 0 of 4 knees). Although these comparisons did not reach statistical significance, the small size of the subgroups, particularly the three‐criterion group, renders them underpowered for formal hypothesis testing, and the reported *p* values should therefore be regarded as exploratory rather than confirmatory. No association was observed between a greater cumulative burden of borderline anatomical abnormalities and poorer outcomes.

### Surgical accuracy and radiographic outcomes

Femoral tunnel positioning was evaluable in 54 of 55 knees, with 31 (57.4%) classified as anatomical and 23 (42.6%) as nonanatomical. The distribution of tunnel positioning did not differ between the standard and borderline groups (*p* = 0.784). No significant differences were observed between anatomical and nonanatomical tunnel positioning in final KOOS (87.3 ± 8.8 vs. 87.4 ± 11.3; *p* = 0.584), Kujala (88.0 ± 10.1 vs. 83.8 ± 18.7; *p* = 0.379), Tegner (4.0 ± 1.1 vs. 3.9 ± 1.3; *p* = 0.426) or VAS scores (1.9 ± 1.7 vs. 2.0 ± 1.3; *p* = 0.515). Redislocation rates were also comparable between anatomical and nonanatomical tunnel positions (2/29 [6.9%] vs. 1/22 [4.5%], respectively; *p* = 1.000). Similarly, distance from the ideal Schöttle point was not significantly correlated with any clinical outcome (all *p* > 0.20).

Postoperative CDI values were comparable between groups, and no significant changes were observed between preoperative and postoperative measurements (mean difference −0.05 ± 0.09; *p* = 0.16).

### Clinical outcomes

All patient‐reported outcome measures improved significantly in both groups from baseline to final follow‐up (*p* < 0.001 for all comparisons).

The mean KOOS improved from 58.0 ± 15.9 preoperatively to 87.3 ± 9.9 at final follow‐up, while the mean Kujala score improved from 57.7 ± 15.5 to 86.2 ± 14.5. The Tegner Activity Scale increased from 1.9 ± 0.9 to 3.9 ± 1.2, and VAS pain scores decreased from 6.4 ± 1.5 to 1.9 ± 1.5.

No statistically significant differences were observed between the standard and borderline groups for any clinical outcome measure at final follow‐up.

Detailed results are reported in Table 3.

**Table 3:** Comparison of the radiological indices and clinical outcomes at baseline and final follow‐up between groups.^a^

	Standard group	Borderline group	*p* value
CDI			
Preoperative	1.03 ± 0.12 (range: 0.81–1.20)	1.15 ± 0.16 (range: 0.80–1.35)	<0.005
Last FU	1.00 ± 0.06 (range: 0.81–1.16)	1.09 ± 0.13 (range: 0.80–1.31)	<0.05
*p* value	0.159	0.159	
KOOS score			
Preoperative	57.30 ± 16.13	58.50 ± 15.91	0.788
Last FU	88.86 ± 10.32	86.13 ± 9.51	0.159
*p* value	<0.0001	<0.0001	
Kujala score			
Preoperative	55.83 ± 17.09	59.07 ± 14.25	0.455
Last FU	85.27 ± 18.83	86.87 ± 10.28	0.713
*p* value	<0.0001	<0.0001	
Tegner score			
Preoperative	1.78 ± 0.74	2.00 ± 0.95	0.579
Last FU	4.00 ± 1.09	3.90 ± 1.27	0.765
*p* value	<0.0001	<0.0001	
VAS score			
Preoperative	6.39 ± 1.47	6.47 ± 1.55	0.858
Last FU	1.61 ± 1.08	2.13 ± 1.78	0.260
*p* value	<0.0001	<0.0001	

Abbreviations: CDI, Caton–Deschamps Index; FU, follow‐up; KOOS, Knee injury Osteoarthritis Outcome Score; SD, standard deviation; VAS, Visual Analogue Scale.

^a^ Data are expressed as mean ± SD. Statistical significance was set at *p* ≤ 0.05.

### Redislocation rate and complications

The overall redislocation rate was 5.4% (3 of 55 knees), with no significant difference between groups (standard group, 4.0%; borderline group, 6.7%; *p* = 1.0). All redislocation events were traumatic and occurred during sports or high‐demand activities.

No intraoperative complications were reported.

In the standard group, two patients developed postoperative stiffness, one requiring manipulation under anaesthesia and one resolving with physiotherapy.

In the borderline group, one patient required removal of a femoral interference screw due to intolerance, one reported persistent hypoesthesia at the graft harvest site, and one experienced transient femoral tunnel pain. Four patients in the borderline group reported persistent anterior knee pain at final follow‐up; all four had undergone MPFL reconstruction using a LARS graft.

## DISCUSSION

The most important finding of the present study is that isolated MPFL reconstruction resulted in comparable mid‐ to long‐term clinical outcomes and redislocation rates in patients with and without borderline anatomical risk factors. Despite a significantly higher burden of preoperative instability in the borderline group, no statistically or clinically relevant differences were observed between groups at a mean follow‐up of nearly 8 years.

In patients presenting with anatomical parameters exceeding normal values but remaining within the borderline ranges examined in this study, these findings suggest that isolated MPFL reconstruction may be a reasonable option to restore patellofemoral stability, without routinely requiring additional bony procedures. This supports a more conservative and individualized surgical strategy, in line with the ‘menu à la carte’ approach [12].

Notably, within the borderline group, cumulative anatomical burden did not predict inferior outcomes: Patients presenting two or three borderline criteria achieved outcomes comparable to those with a single criterion, with no increase in redislocation rate. This finding supports the concept that the decision to add bony procedures should be driven by the severity of individual parameters rather than their number alone.

The present study is among the longest follow‐up investigations directly comparing outcomes of isolated MPFL reconstruction in patients with and without predisposing anatomical risk factors. Long‐term data are particularly relevant in patellofemoral instability, as recurrence, persistent pain and cartilage degeneration may occur several years after surgery. The durability of the clinical improvements observed in both groups reinforces the role of MPFL reconstruction as a reliable stabilizing procedure when appropriate indications and surgical technique are applied.

The effectiveness of isolated MPFL reconstruction in treating recurrent patellar instability has been widely demonstrated in the literature [14, 15], with consistently low redislocation rates [41] and significant improvements in patient‐reported outcomes [17, 33]. Long‐term follow‐up studies [31, 43] have further confirmed the ability of MPFL reconstruction to provide sustained patellar stability by restoring the primary passive restraint to lateral patellar translation. The present results are consistent with these findings, as both groups showed significant improvements in KOOS, Kujala, Tegner and VAS scores, with no between‐group differences at final follow‐up.

A relevant aspect of the present cohort is the higher number of preoperative dislocation episodes in the borderline group. Previous studies have suggested that recurrent instability may negatively affect postoperative outcomes [36]. However, in the present study, a higher preoperative instability burden did not translate into worse results. This suggests that the number of prior dislocations alone should not necessarily dictate a more aggressive surgical strategy when anatomical risk factors remain within borderline ranges.

The management of borderline anatomical risk factors remains one of the most debated aspects in patellar instability surgery. While the indication for additional bony procedures is relatively clear in the presence of severe abnormalities, decision‐making becomes more complex when anatomical parameters fall within intermediate ranges. This often leads to variability in surgical indications across centres and surgeons. Recent consensus statements [3] emphasize individualized decision‐making but acknowledge the lack of high‐quality evidence defining clear thresholds for intervention in these patients [9, 32, 37].

In the present study, borderline anatomy was defined using specific quantitative ranges for patellar height and TT–TG distance, together with the presence of trochlear dysplasia. This approach reflects a more functional interpretation of anatomical risk factors, considering not only their presence but also their magnitude. The results suggest that mild to moderate deviations from normal values do not necessarily compromise the effectiveness of isolated MPFL reconstruction when appropriate patient selection is applied.

Trochlear dysplasia deserves particular attention. Although the borderline group included a substantial proportion of patients with higher‐grade dysplasia (Types B–D), no differences in clinical outcomes or redislocation rates were observed. This finding supports the concept that trochlear morphology alone should not be considered an absolute indication for trochleoplasty in the absence of clearly pathological features. Recent evidence suggests that the stabilizing effect of MPFL reconstruction may partially compensate for moderate trochlear abnormalities [25, 29], particularly when dynamic tracking is adequately restored.

Similarly, patellar height did not negatively influence outcomes when values remained within borderline ranges. Although the borderline group exhibited higher CDI values, no inferior clinical results were observed. Previous studies have reported a spontaneous reduction in CDI following MPFL reconstruction [21, 27, 39], likely related to improved patellar tracking and soft tissue balance. Although the reduction observed in the present study did not reach statistical significance, the absence of inferior outcomes supports the notion that tibial tubercle distalization should be reserved for clearly pathological patella alta rather than borderline values.

The assessment of SPE provides additional insight into functional patellofemoral mechanics. While SPE values were slightly lower in the borderline group, mean values remained within the normal range in both groups. According to the functional concept proposed by Dejour et al. [13], patellar height and engagement should be interpreted along a continuum rather than as dichotomous variables. In this framework, borderline reductions in SPE may reflect a diminished functional reserve without fulfilling criteria for functional patella alta. This may explain why higher CDI values and more complex trochlear morphology did not translate into inferior outcomes following isolated MPFL reconstruction.

The same applies to TT–TG distance. Moderate increases did not adversely affect clinical outcomes in the present cohort, reinforcing the concept that TT–TG should not be considered in isolation but rather interpreted within the overall anatomical and functional context [15, 17].

Beyond patient selection, surgical technique remains an important determinant of MPFL reconstruction outcomes. Although 42.6% of evaluable knees had a nonanatomical femoral tunnel position, tunnel positioning was not associated with inferior clinical outcomes or redislocation and was similarly distributed between the standard and borderline groups. Likewise, distance from the ideal Schöttle point was not correlated with clinical outcomes. These findings suggest that tunnel positioning was unlikely to substantially confound the primary between‐group comparison, although the relatively small sample size warrants cautious interpretation.

Complication rates were low and comparable to those reported in the literature. Notably, the four patients reporting persistent anterior knee pain were among the 10 patients in the borderline group treated with a LARS graft. Although no definitive conclusions can be drawn from such a small number of cases, this observation is consistent with the greater stiffness of synthetic grafts and suggests that graft choice may influence postoperative patellofemoral biomechanics, particularly in patients with borderline anatomical risk factors.

Overall, the present study supports a more conservative surgical approach in carefully selected patients with borderline anatomical risk factors. Rather than representing undertreatment, isolated MPFL reconstruction may provide an optimal balance between surgical invasiveness and clinical effectiveness.

## LIMITATIONS

This study has several limitations. Its retrospective design introduces inherent selection bias. The sample size was relatively small, although sufficient to detect clinically meaningful differences between groups. In addition, multiple between‐group and within‐group comparisons were performed without adjustment for multiple testing. Although the analyses were based on predefined primary outcomes, this may have increased the risk of Type I error, and therefore secondary findings should be interpreted with appropriate caution. Radiological assessment was performed using different imaging modalities, reflecting clinical practice but introducing some heterogeneity. Additionally, clinical parameters such as the J‐sign and apprehension test were not quantitatively standardized. Finally, cartilage status was not systematically assessed arthroscopically.

Furthermore, coronal and rotational alignment were not systematically evaluated. Full lower‐limb CT scans were not routinely performed, and in many cases only knee CT was available, which does not allow reliable assessment of femoral and tibial rotational deformities. Therefore, the potential influence of coronal and rotational abnormalities on clinical outcomes could not be fully analysed.

Finally, the conclusions of this study apply only within the range of anatomical values actually included: patellar height up to a CDI of approximately 1.35, TT–TG distance up to 24 mm and Dejour Type B, C or D trochlear dysplasia in the absence of an explosive J‐sign or a large trochlear spur. Whether isolated MPFL reconstruction remains sufficient beyond these values, in the presence of clearly pathological patella alta or higher TT–TG distances, cannot be inferred from the present data and may still warrant additional bony procedures.

## STRENGTHS

The strengths of this study include the relatively long follow‐up, the use of clearly defined inclusion criteria and the direct comparison between patients with and without borderline anatomical risk factors. Furthermore, the use of quantitative thresholds to define borderline anatomy improves the reproducibility and clinical applicability of the findings.

## CONCLUSION

Isolated MPFL reconstruction provides reliable and durable mid‐ to long‐term outcomes in carefully selected patients with borderline anatomical risk factors, with clinical results and redislocation rates comparable to those observed in standard patients. These findings support a patient‐specific surgical approach, suggesting that additional bony procedures should be reserved for clearly pathological anatomical abnormalities rather than routinely performed in borderline cases.

## AUTHOR CONTRIBUTIONS

**Alessio Maione**: Conceptualization; study design; surgical procedures; manuscript revision. **Simone Romano**: Patient recruitment; study design; data analysis; statistical analysis; manuscript drafting **Pierrenzo Pozzi**: Patient recruitment; data analysis; critical revision. **Matteo D. Parmigiani**: Methodology supervision; interpretation of data; final approval. **Alessandra Menon**: Statistical analysis. **Paolo Ferrua**: Surgical procedures; methodology supervision; final approval. **Filippo Calanna**: Methodology supervision; interpretation of data. **Riccardo Compagnoni**: Manuscript revision; critical revision. **Eva Usellini**: Data analysis; patient recruitment. **Andrea Mambretti**: Interpretation of data; study design. **Massimo Berruto**: Surgical procedures; conceptualization; methodology supervision; final approval. **Pietro S. Randelli**: Methodology supervision; final approval.

## FUNDING INFORMATION

The authors have no funding to report.

## CONFLICT OF INTEREST STATEMENT

The authors declare no conflicts of interest.

## ETHICS STATEMENT

This study was approved by Milano Area 2—Fondazione IRCCS Ca' Granda Ospedale Maggiore Policlinico, Milan, Italy; approval ID: 870_2021.

## Supporting information

Supporting File 1.

## Data Availability

The data that support the findings of this study are available from the corresponding author upon reasonable request.
